# Analysis of Cases Under the Protection of Children From Sexual Offences (POCSO) Act at a Tertiary Care Hospital: A Retrospective Descriptive Study From Chennai

**DOI:** 10.7759/cureus.116814

**Published:** 2026-09-24

**Authors:** Vanitha V, Shanthi S, Jebakkani J, Anusuya P

**Affiliations:** 1 Department of Obstetrics and Gynecology, Government Kilpauk Medical College and Hospital, Chennai, IND

**Keywords:** adolescent, child marriage, child sexual abuse, elopement, medical termination of pregnancy, pocso, social media grooming, term pregnancy

## Abstract

Background and aim

Child sexual abuse is a significant public health crisis globally. Despite mandatory reporting requirements under the Protection of Children from Sexual Offences (POCSO) Act, systematic hospital-based data on the clinical and obstetric profiles of POCSO victims from individual tertiary care centers in Chennai remain limited. This study aimed to describe the clinico-socioeconomic profile, mode of communication, violence, pregnancy outcome, and medical termination of pregnancy (MTP) in POCSO cases, with a specific focus on victims aged under 18 years presenting with a continuing pregnancy.

Materials and methods

A hospital-based retrospective descriptive study was conducted at Government Kilpauk Medical College and Hospital, Chennai. Data were extracted from the accident register or AR book for all POCSO cases recorded from January 2025 to June 2026 (18 months). No patient consent was required as this was a record-based retrospective study. A total of 250 cases were analyzed.

Results

The mean victim age was 15.2 years, and 60.0% were aged 16-17 years. Based on socioeconomic classification, 80.8% belonged to the lower middle class. Social media was the predominant grooming channel (57.6%), with Instagram (27.6%) and WhatsApp (23.6%) (Menlo Park, CA: Meta Platforms, Inc.) leading. Elopement was documented in 45.6%. Juvenile alleged offenders constituted 23.2%. Violence was present in 9.2%; physical injuries in 5.2%. Pregnancy was detected in 27.6% (n=69). Among pregnant victims, 56.5% (n=39) initially expressed willingness to continue the pregnancy, all aged 14-18 years, predominantly from the 16-17 year group (71.8%), 87.2% from the lower middle class, with 89.7% having no contraception awareness. Overall, MTP was subsequently performed in 88.4% (n=61) of pregnant victims, with 85.2% of terminations occurring between 12 and 20 weeks of gestation. Willingness to continue pregnancy was recorded at the time of initial assessment and represented the patient's stated preference at presentation; it did not necessarily correspond to the subsequent pregnancy outcome.

Conclusion

The subgroup of pregnant victims who wished to continue the pregnancy constituted a distinct medico-legal group requiring multidisciplinary co-management. Digital literacy, reproductive health education, and child marriage prevention are essential priorities.

## Introduction

Child sexual abuse (CSA) is a grave violation of child rights and a global public health crisis. The World Health Organization (WHO) estimates that one in five women and one in 13 men report having been sexually abused during childhood [1]. In India, the Protection of Children from Sexual Offences (POCSO) Act, 2012 provides a comprehensive legal framework to protect persons under 18 years from sexual assault, harassment, and pornography, with provision for special courts for expedited trial [2].

The National Crime Records Bureau (NCRB) recorded 62,095 cases under the POCSO Act in 2022, representing an 8.7% increase in crimes against children compared to 2021 [3]. Despite mandatory reporting requirements under Rule 5 of the POCSO Act, which obligates all medical professionals to provide emergency care to any POCSO victim presenting at their facility, systematic hospital-based data on clinical and obstetric profiles of victims remain sparse in South India [4].

In this study, grooming was operationally defined as a process in which an alleged perpetrator establishes contact, trust, emotional connection, or manipulative interaction with a minor with the purpose of facilitating sexual exploitation; social media contact alone was not considered sufficient to classify an interaction as grooming. Instagram, WhatsApp (Menlo Park, CA: Meta Platforms, Inc.), and other digital platforms are increasingly used by perpetrators to establish contact with minors [5]. A clinically distinct and under-reported subgroup is emerging, i.e., adolescent girls under 18 years who elope with age-similar peers, conceive, and present at tertiary hospitals at term or near-term gestation wishing to continue the pregnancy. This group sits at the complex medico-legal intersection of the POCSO Act, the Prohibition of Child Marriage Act, 2006, and obstetric care.

The Medical Termination of Pregnancy (MTP) (Amendment) Act 2021 extended termination provisions for rape survivors up to 24 weeks [6]. However, victims who wish to continue pregnancy under POCSO present an equally complex clinical and legal challenge. The present study aimed to describe the clinico-socioeconomic profile, mode of communication, violence, pregnancy outcomes, and medical termination of pregnancy (MTP) among POCSO cases presenting to a tertiary care hospital in Chennai.

## Materials and methods

Study design, setting, period, and sample size

A hospital-based retrospective descriptive study was conducted in the Department of Obstetrics and Gynecology, Government Kilpauk Medical College and Hospital, Chennai, Tamil Nadu, India. Data were extracted from the accident register, or AR book, of the Department of Obstetrics and Gynecology for all POCSO cases recorded from January 2025 to June 2026 (18 months). All eligible cases registered in the AR book during this period were included; no formal sample size calculation was applied, as the study is a census of all POCSO records available during the study period. A total of 250 POCSO cases were identified, and all 250 cases were included in the final analysis.

Ethical considerations

The Institutional Ethics Committee of Government Kilpauk Medical College, Chennai, granted ethical approval (#18/2026) for the retrospective use of medico-legal records from the accident register. The IEC approved a waiver of individual informed consent (and assent, if applicable), as the study involved retrospective review of existing records and the data were anonymized before analysis. Patient confidentiality was maintained throughout the study. As this retrospective, record-based study used data from institutional registers with no patient identifiers, individual patient consent and assent were not required per the Indian Council of Medical Research (ICMR) National Ethical Guidelines for Biomedical and Health Research Involving Human Participants, 2017.

Inclusion and exclusion criteria

All medico-legal cases registered under the POCSO Act, 2012 and recorded in the AR book of the Department of Obstetrics and Gynecology during the study period were included. Cases with incomplete records lacking core variables (age, pregnancy status, or mode of contact) were excluded.

Data collection

Data were extracted from AR book entries onto a predesigned structured proforma capturing as follows: victim demographics and socioeconomic status by Kuppuswamy classification [7]; family background including family type, parental status, and both parents' working status; alleged offender profile including age and juvenile status per the Juvenile Justice Act, 2015; mode of communication; place of sexual act; elopement status; violence and physical injury; contraception awareness; pregnancy status; willingness to continue pregnancy; and gestational age at MTP where applicable [8].

A structured proforma was developed specifically for this study based on the study objectives and variables available in the accident register. The proforma was pilot-tested on a small set of records before the main data extraction to ensure clarity and completeness. Data extraction was performed by two investigators, with discrepancies resolved by discussion and consensus.

For retrospective ascertainment, grooming was recorded when the accident register documentation described a pattern of establishing contact, trust, emotional attachment, or manipulation with the minor for the purpose of sexual exploitation. Mere contact through social media or other communication platforms was not classified as grooming in the absence of such documentation.

Age was recorded as the age of the victim at the time of hospital presentation/medical assessment. The age at the time of the alleged offence was also reviewed where available in the medico-legal records. All three participants who were aged 18 years at hospital presentation were also 18 years or older at the time of the alleged offence and therefore were not classified as under-18-year-old victims.

Statistical analysis

Data were analyzed using Microsoft Excel 2021 (Redmond, WA: Microsoft Corp.). Descriptive statistics are reported as frequencies and percentages. Cross-tabulation was performed for selected variables. Figures were generated using Python 3.13.0 (Matplotlib 3.9.2; Matplotlib Development Team). All data are presented as frequency tables and charts.

## Results

Socioeconomic profile of victims

A total of 250 POCSO cases were retrieved from the AR book from January 2025 to June 2026. The mean age of victims was 15.2±1.8 years (range: 4-18 years). The 16 to 17-year age group was the most affected, comprising 60.0% (n=150) of cases, followed by 14-15 years (26.4%; n=66). Preschool children below 10 years constituted 6.4% (n=16) (Table 1, Figure 1) [7]. These proportions are consistent with National Crime Records Bureau (NCRB) 2022 data and published in the South Bangalore series [3]. In terms of occupation, 93.6% (n=234) of victims were school-going students, and 6.4% (n=16) were pre-school children. The lower middle class comprised 80.8% (n=202) of cases, reflecting the heightened vulnerability of economically disadvantaged households.

**Table 1:** Clinico-socioeconomic profile of POCSO victims (n=250). SES: socioeconomic status; LMC: lower middle class; UMC: upper middle class; POCSO: Protection of Children from Sexual Offences

Variables	Category	n	%
Age of victim (years)	<10	16	6.4
	10-13	15	6
	14-15	66	26.4
	16-17	150	60
	18	3	1.2
Mean age (years)	15.2±1.8	-	-
Victim occupation	School-going students (≥10 years)	234	93.6
	Preschool child (<10 years)	16	6.4
Socioeconomic status	Lower middle class	202	80.8
Kuppuswamy classification	Upper middle class	48	19.2
Total	-	250	100

**Figure 1:**
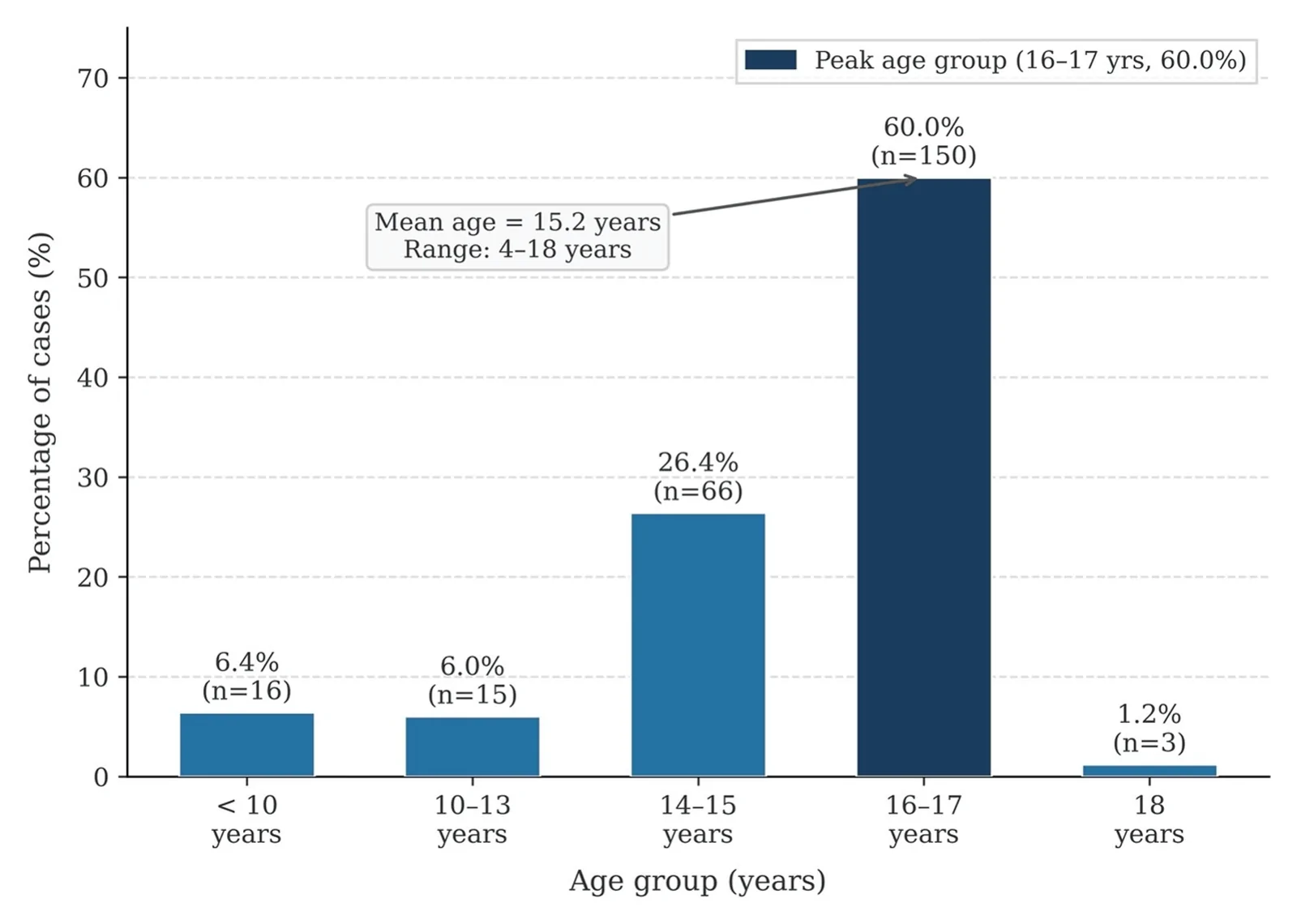
Age group distribution of POCSO victims (n=250). POCSO: Protection of Children from Sexual Offences

Family background and parental status

Disrupted or broken family background was identified in 43.6% (n=109) of victims' families (Table 2). Among victims, 18.4% (n=46) had both parents employed outside the home, representing unsupervised home environments as a key risk factor. A comparable pattern was observed in alleged offenders' families (both parents working: 17.6%; n=44). Parental unavailability and family instability are established risk factors for child sexual abuse (CSA) [9].

**Table 2:** Family background and parental status of victims and alleged offenders (n=250).

Variables	Category	n	%
Family type	Intact family	141	56.4
	Disrupted/broken family	109	43.6
Parental status - victim	Living together	141	56.4
	Both parents working	46	18.4
	Separated	33	13.2
	Divorced	17	6.8
	Single parent	10	4
	Orphan	3	1.2
Both parents working - victim	Yes	46	18.4
Parental status - alleged offender	Living together	164	65.6
	Both parents working	44	17.6
	Separated	21	8.4
	Divorced	14	5.6
	Single parent/orphan	7	2.8
Both parents working - alleged offender	Yes	44	17.6
Total		250	100

Profile of alleged offenders

Young adults in the 18-30-year group constituted the largest alleged offender cohort (69.2%; n=173). Crucially, 23.2% (n=58) of alleged offenders were themselves juveniles (<18 years), indicating a significant peer-perpetration pattern (Table 3, Figures 2A, 2B). This finding has direct implications for the interface between the POCSO Act and the Juvenile Justice (Care and Protection of Children) Act 2015 [8].

**Table 3:** Profile of alleged offenders - age and juvenile status (n=250).

Variables	Category	n	%
Age group (years)	<18 (juvenile)	58	23.2
	18-30	173	69.2
	30-60	7	2.8
	>60	9	3.6
	Not known/not recorded	3	1.2
Juvenile status	Juvenile (<18 years) - peer perpetration	58	23.2
	Adult (≥18 years)	192	76.8
Total		250	100

**Figure 2:**
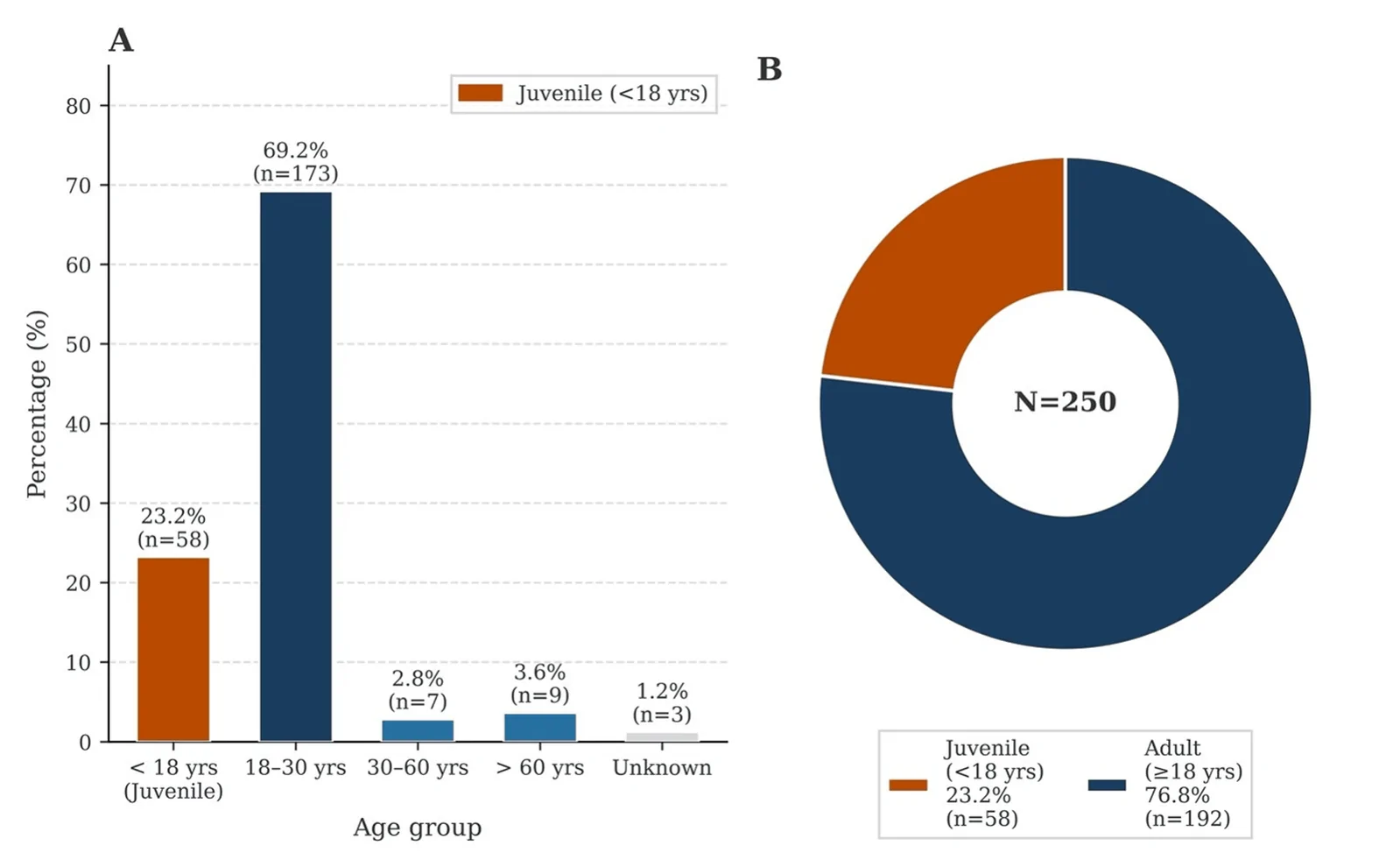
Age group distribution of alleged offenders and juvenile vs. adult proportion (n=250).

Mode of communication and grooming

Social media platforms accounted for 57.6% (n=144) of all grooming contacts (Table 4, Figure 3). Instagram was the most frequently used platform (27.6%; n=69), followed by WhatsApp (23.6%; n=59) and Facebook (Menlo Park, CA: Meta Platforms, Inc.) (4.4%; n=11). School contact accounted for 8.8% (n=22) and neighbor or relative contact for 11.2% (n=28). The remaining 7.2% (n=18) comprised mobile calls, park or public place contacts, and unclassified modes of contact. The dominance of Instagram as a grooming platform is a novel finding from this South Indian hospital series, consistent with global evidence on technology-facilitated child sexual exploitation [10]. These findings support urgent policy intervention through mandatory digital literacy programs in school curricula and regulatory obligations for social media platforms operating in India.

**Table 4:** Mode of communication between victim and alleged offender (n=250). Social media subtotal: Instagram + WhatsApp + Facebook (Menlo Park, CA: Meta Platforms, Inc.) + dating app+online game = 144 (57.6%). Not recorded/unclassified includes not recorded (n=10), mobile call (n=4), park/public place (n=2), and not applicable (n=2) = 18 cases. The frequencies across all categories in the table represent the complete study cohort of 250 POCSO cases. POCSO: Protection of Children from Sexual Offences

Mode of communication	n	%	Category
Instagram	69	27.6	Social media
WhatsApp	59	23.6	Social media
Facebook	11	4.4	Social media
Dating app/online game	5	2	Social media
Social media subtotal	144	57.6	-
School contact	22	8.8	In-person
Mutual friend/acquaintance	17	6.8	In-person
Neighbor	14	5.6	In-person
Relative	14	5.6	In-person
Stranger/workplace/other	21	8.4	Other
Not recorded	18	7.2	-
Total	250	100	-

**Figure 3:**
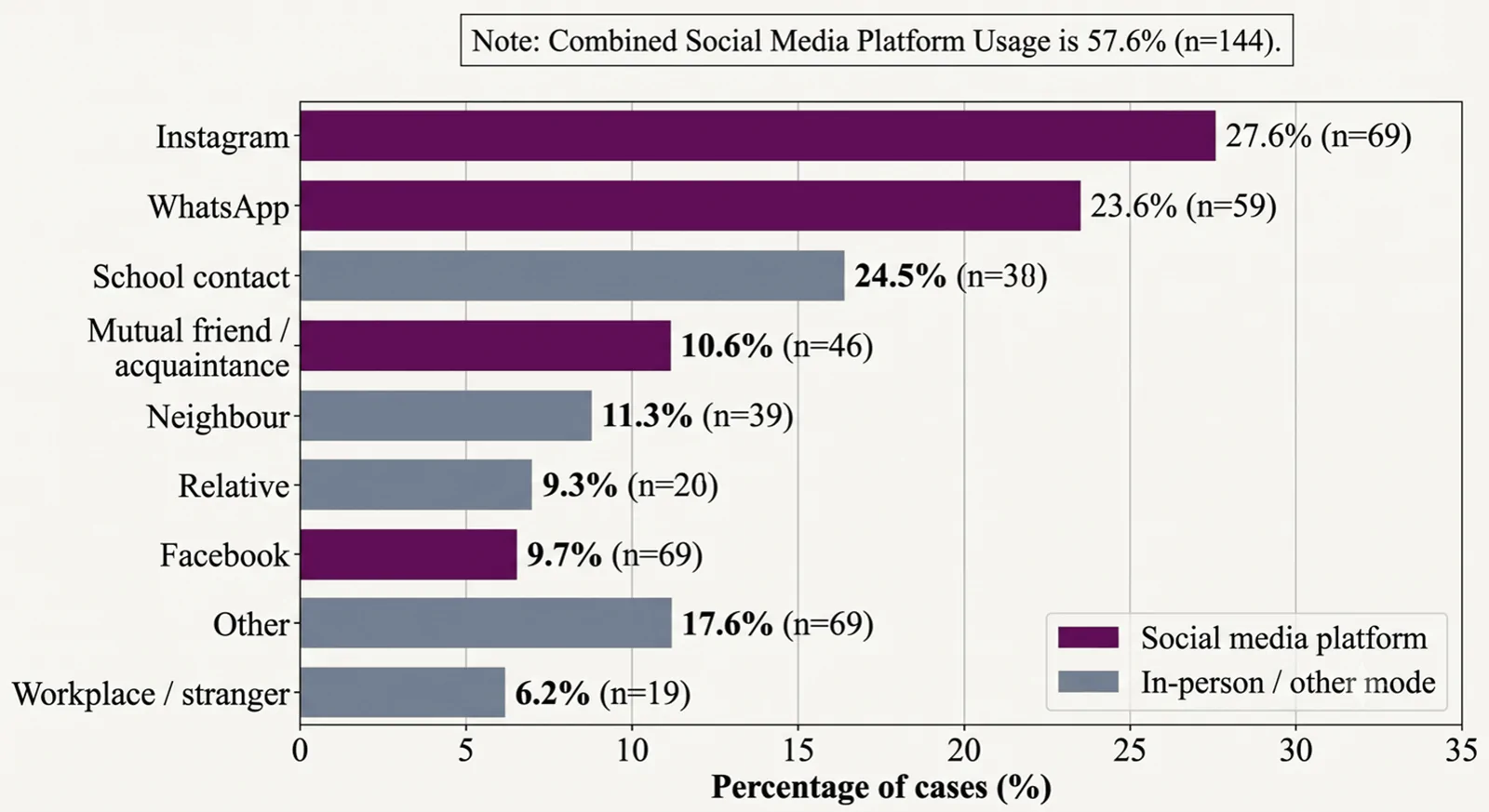
Mode of communication between victim and alleged offender (n=250). Instagram, WhatsApp, Facebook (Menlo Park, CA: Meta Platforms, Inc.)

Elopement, place of offence, violence, and contraception

Elopement was documented in 45.6% (n=114) of cases (Table 5, Figures 4A, 4B). A total of 74.4% (n=186) of offences occurred in familiar private settings - victim's home (n=82; 32.8%), alleged offender's home (n=79; 31.6%), or relative's home (n=25; 10.0%). Rented hotels or lodges accounted for 9.6% (n=24). Physical violence or coercion was documented in only 9.2% (n=23) of cases, and external injuries in 5.2% (n=13); however, the retrospective records do not permit determination of legally valid consent or the absence of coercion. Violence rates were similar in eloped (n=10; 8.8%) and non-eloped (n=13; 9.6%) groups, confirming that absence of elopement does not imply physical coercion. These rates are consistent with the Indian POCSO literature [5]. Contraception awareness was absent in 88.4% (n=221) of victims, with condom use in only 11.6% (n=29). Among those who eloped, 89.5% (n=102 of 114) had nil contraception awareness, directly accounting for the high pregnancy rate of 27.6% (n=69).

**Table 5:** Elopement, place of offence, violence, injury, and contraception awareness (n=250). *Percentage calculated within elopement subgroup (eloped n=114; non-eloped n=136).

Variables	Category	n	%
Elopement	Yes	114	45.6
	No	136	54.4
Place of sexual act	Victim's home	82	32.8
	Alleged offender's home	79	31.6
	Relative's home	25	10
	Rented hotel/lodge	24	9.6
	Others	40	16
Familiar private setting (subtotal)		186	74.4
Violence/physical coercion	Present	23	9.2
Physical injuries	Present	13	5.2
Contraception awareness	Nil/none	221	88.4
	Condom used	29	11.6
Violence - eloped (n=114)	Present	10	8.8*
Violence - non-eloped (n=136)	Present	13	9.6*
Total		250	100

**Figure 4:**
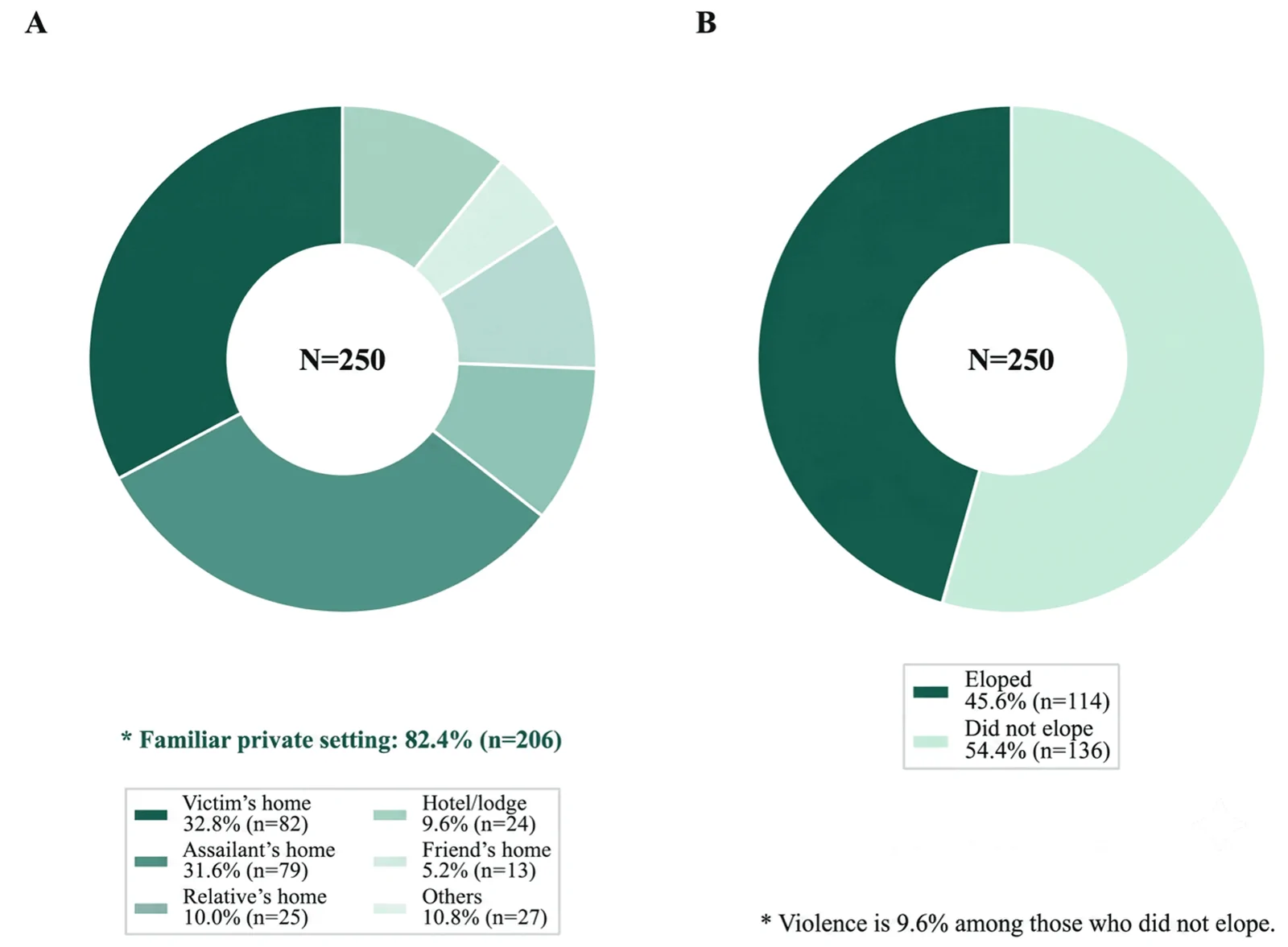
Elopement status and place of sexual act (n=250).

Obstetric outcomes and MTP

Pregnancy was detected in 27.6% (n=69) of the 250 victims (Table 6, Figures 5A, 5B). Three victims (1.2%) were pre-menarche at examination. Among pregnant victims, 56.5% (n=39) initially expressed willingness to continue the pregnancy. Overall, MTP was subsequently performed in 88.4% (n=61) of pregnant victims. The gestational age at MTP was predominantly between 12 and 20 weeks as follows: 85.2% (n=52) of MTPs were performed between 12 and 20 weeks, corresponding to Section 3(2)(b) of the MTP Act; 9.8% (n=6) were performed between 20 and 24 weeks under Section 3(2B) for rape survivors; and 4.9% (n=3) were performed before 12 weeks [6]. The predominance of second-trimester MTP reflects a systemic pattern of delayed presentation, encompassing delays in case registration, forensic examination, and obstetric referral, and demands fast-track MTP referral protocols at all government hospitals receiving POCSO cases.

**Table 6:** Obstetric outcomes and gestational age at medical termination of pregnancy (n=250). *% within pregnant subgroup (n=69). **% within MTP subgroup (n=61). Sec: Section of the MTP (Amendment) Act, 2021; MTP: medical termination of pregnancy

Variables	Category	n	%
Pregnancy status (n=250)	Pregnant	69	27.6
	Not pregnant	172	68.8
	Pre-menarche	3	1.2
	Not recorded	6	2.4
Willing to continue (n=69)	Yes	39	56.5*
	No (sought MTP)	22	31.9*
	Not recorded	8	11.6*
MTP performed (n=69)	Yes	61	88.4*
	No (continued pregnancy)	8	11.6*
Gestational age at MTP (n=61)	<12 weeks - Sec 3(2)(a)	3	4.9**
	12-20 weeks - Sec 3(2)(b) (predominant)	52	85.2**
	20-24 weeks - Sec 3(2B) rape survivor	6	9.8**
	>24 weeks - judicial/board order	0	0**
Total		250	-

**Figure 5:**
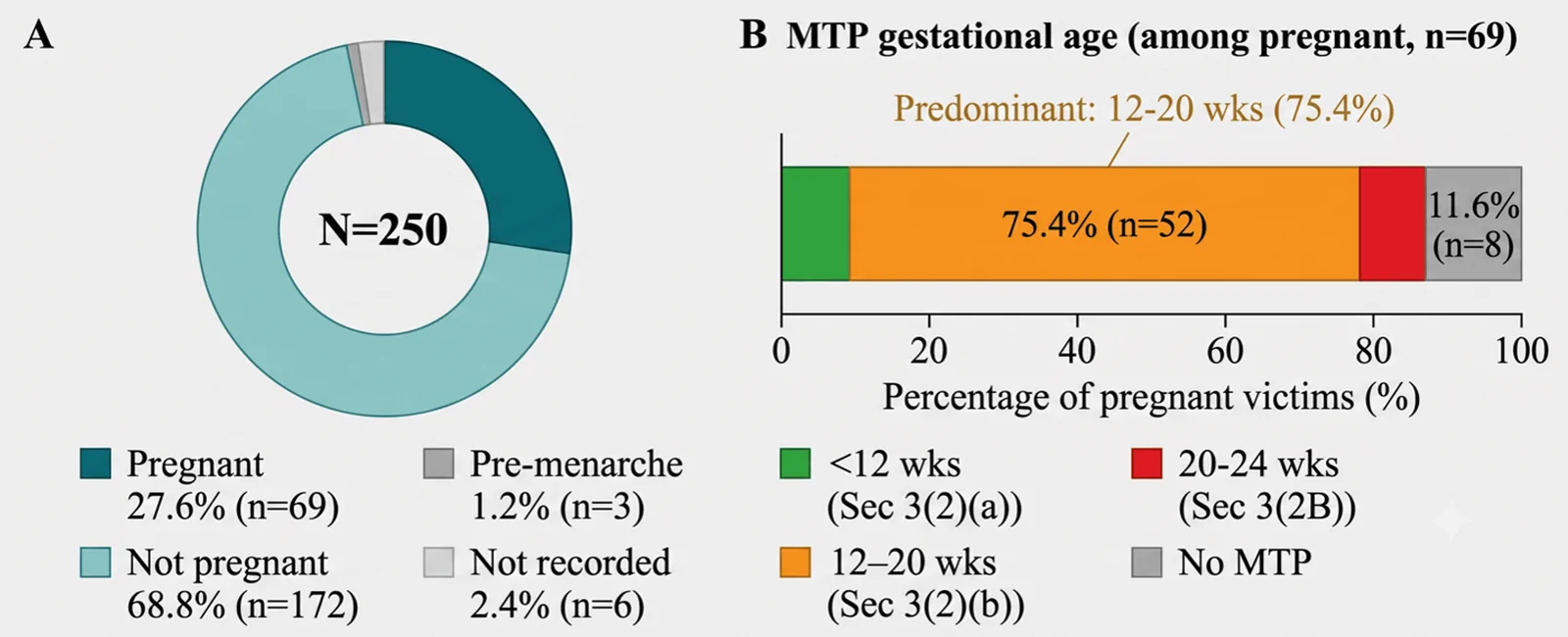
Pregnancy status and gestational age at MTP among POCSO victims (n=250). MTP: medical termination of pregnancy; POCSO: Protection of Children from Sexual Offences

Pregnant POCSO victims who initially wished to continue pregnancy - a distinct clinical subgroup

A clinically and medico-legally relevant subgroup comprised 39 pregnant victims (56.5% of all pregnant cases; 15.6% of the total cohort of 250) who initially expressed willingness to continue the pregnancy and presented at near-term or term gestation. Table 7 summarizes their characteristics.

**Table 7:** Profile of pregnant POCSO victims who initially expressed willingness to continue pregnancy (n=39; 56.5% of pregnant subgroup). LMC: lower middle class; UMC: upper middle class; POCSO: Protection of Children From Sexual Offences

Characteristics	Finding (n)	% of subgroup (n=39)
Elopement prior to pregnancy	Yes (21), no (18)	53.8% eloped, 46.2% non-eloped
Age group	16-17 years (28)	71.8%
	14-15 years (10)	25.6%
	18 years (1)	2.6%
Socioeconomic status	LMC (34)	87.2% lower middle class
	UMC (5)	12.8% upper middle class
Violence documented	1 case	2.6% - physical violence documented in one case
Contraception awareness	Nil (35)	89.7% - no contraception used
Alleged offender age group	Predominantly <18 or 18-25 years	Peer-aged/young adult relationship reported in records

All 39 victims in this subgroup were between 14 and 18 years of age. Of these, 38 were below 18 years of age, and one was 18 years of age at the time of the alleged offence. The 16-17 year group predominated (71.8%; n=28), followed by 14-15 years (25.6%; n=10) and 18 years (2.6%; n=1). Elopement preceded the pregnancy in 53.8% (n=21). Only one case (2.6%) had documented physical violence, and 89.7% (n=35) had no contraception awareness. These cases were predominantly characterized in the available records as adolescent relationships involving unprotected sexual activity; however, the retrospective records do not permit assessment of legally valid consent under the POCSO Act. Some cases involved adolescent relationships with circumstances suggestive of cohabitation or child marriage.

Lower middle-class SES was the predominant background (87.2%; n=34). Alleged offenders were predominantly peer-aged (juvenile or young adult). These cases sit at the complex medico-legal intersection of the POCSO Act, the Prohibition of Child Marriage Act, 2006, POCSO Rules, 2020 (Rule 5 - best interests of the child), and obstetric care protocols for adolescent pregnancy [2].

These cases present a significant clinical challenge. The victim, though legally a child, expresses autonomous reproductive intent, often supported by family. Multidisciplinary management involving obstetrics, pediatrics, forensic medicine, social welfare, and legal counsel is essential. Obstetric risks in this group include anemia, preeclampsia, obstructed labor, low birth weight, and neonatal complications.

From a policy perspective, the POCSO Act criminalizes all sexual activity involving persons under 18 regardless of consent, yet the Prohibition of Child Marriage Act does not automatically void child marriages, and the POCSO Rules, 2020 simultaneously mandate the best interests of the child as the paramount consideration. This legislative paradox requires institutional guidelines and clinical protocols.

## Discussion

Table 8 compares key findings with the published Indian POCSO series. The elopement rate (45.6%), age distribution (peak: 16-17 years; 60.0%), and social media grooming proportion (57.6%) from the present n=250 study are broadly consistent with or exceed published rates. The specifically highlighted subgroup of continuing pregnancy in under-18-year-old victims (n=39; 56.5% of pregnant cases; 15.6% of all cases) is a novel finding not previously described as a discrete clinical subgroup in South Indian hospital-based POCSO literature.

**Table 8:** Comparison of key findings with published Indian POCSO studies (present study n=250). NCRB: National Crime Records Bureau; POCSO: Protection of Children From Sexual Offences; NE: Northeast India

Variables	Present study (n=250, Chennai, January 2025-June 2026)	Sujatha et al. (2020) (Bangalore) [5]	Sharma et al. (2025) (NE India) [11]	NCRB 2022 [3]
Peak victim age	16-17 years (60.0%)	13-18 years	13-18 years	<18 years
Social media grooming	57.6% - novel	Not reported	Not reported	Not reported
Elopement	45.6%	Not reported	Reported; 18/48 (37.5%) among cases described as consensual	Common
Juvenile alleged offenders	23.2%	Not specified	Not presented in this form	Increasing
Violence/injury	9.2%/5.2%	Not specified	Injury present in 14/48 (29.17%)	Variable
Pregnancy rate	27.6% - novel	Not reported	3/48 (6.25%)	Not reported
Continuing pregnancy (<18 years)	39 cases (56.5% of pregnant) - novel	Not reported	Not reported	Not reported

Limitations

This study relies on retrospective accident register data, which may under-record nuanced variables such as alleged offender occupation and precise clinical circumstances. Only reported cases are captured; therefore, the findings may not represent the true burden of child sexual abuse in the community. The retrospective nature of the study also limits the ability to reliably determine the circumstances surrounding willingness to continue pregnancy and other contextual factors. As this was a single-center retrospective study, the findings should be interpreted within the context of the study hospital and should not be generalized to the wider population of South India.

## Conclusions

This retrospective descriptive study of 250 POCSO cases at a South Indian tertiary center over 18 months reveals that adolescent girls aged 16-17 years from lower middle class, disrupted family backgrounds constitute the dominant victim profile. Social media, principally Instagram (27.6%) and WhatsApp (23.6%), accounted for 57.6% of all grooming contacts, a novel and policy-relevant finding from South India.

A critically important and previously under-reported subgroup emerged as follows: 39 pregnant victims (56.5% of pregnant cases; 15.6% of the total cohort) who wished to continue the pregnancy and presented at near-term or term gestation. This group constitutes a distinct clinical and medico-legal entity requiring multidisciplinary obstetric, social, and legal co-management, and highlights the complex legal and clinical considerations surrounding adolescent pregnancy following sexual abuse. Priority interventions include mandatory digital literacy and reproductive health education from the school level, adolescent-friendly obstetric protocols for pregnancies in victims under 18 years of age, institutional guidelines for POCSO cases presenting with continuing pregnancy, and timely MTP referral pathways to facilitate access to legally permissible termination when requested.
